# Structure-Based Virtual Screening to Identify Novel Potential Compound as an Alternative to Remdesivir to Overcome the RdRp Protein Mutations in SARS-CoV-2

**DOI:** 10.3389/fmolb.2021.645216

**Published:** 2021-04-09

**Authors:** Thirumal Kumar D, Nishaat Shaikh, Udhaya Kumar S, George Priya Doss C, Hatem Zayed

**Affiliations:** ^1^Meenakshi Academy of Higher Education and Research, Chennai, India; ^2^School of Bio Sciences and Technology, Vellore Institute of Technology, Vellore, India; ^3^Saveetha School of Engineering, Saveetha Institute of Medical and Technical Sciences, Chennai, India; ^4^Department of Biomedical Sciences, College of Health and Sciences, Qatar University, QU Health, Doha, Qatar

**Keywords:** SARS-CoV-2, COVID-19, remdesivir, virtual screening, RdRp, mutations

## Abstract

The number of confirmed COVID-19 cases is rapidly increasing with no direct treatment for the disease. Few repurposed drugs, such as Remdesivir, Chloroquine, Hydroxychloroquine, Lopinavir, and Ritonavir, are being tested against SARS-CoV-2. Remdesivir is the drug of choice for Ebola virus disease and has been authorized for emergency use. This drug acts against SARS-CoV-2 by inhibiting the RNA-dependent-RNA-polymerase (RdRp) of SARS-CoV-2. RdRp of viruses is prone to mutations that confer drug resistance. A recent study by Pachetti et al. in 2020 identified the P323L mutation in the RdRp protein of SARS-CoV-2. In this study, we aimed to determine the potency of lead compounds similar to Remdesivir, which can be used as an alternative when variants of SARS-CoV-2 develop resistance due to RdRp mutations. The initial screening yielded 704 compounds that were 90% similar to the control drug, Remdesivir. On further evaluation through drugability and antiviral inhibition percentage analyses, we shortlisted 32 and seven compounds, respectively. These seven compounds were further analyzed for their molecular interactions, which revealed that all seven compounds interacted with RdRp with higher affinity than Remdesivir under native conditions. However, three compounds failed to interact with the mutant protein with higher affinity than Remdesivir. Dynamic cross-correlation matrix (DCCM) and vector field collective motions analyses were performed to identify the precise movements of docked complexes' residues. Furthermore, the compound SCHEMBL20144212 showed a high affinity for native and mutant proteins and might provide an alternative against SARS-CoV-2 variants that might confer resistance to Remdesivir. Further validations by *in vitro and in vivo* studies are needed to confirm the efficacy of our lead compounds for their inhibition against SARS-CoV-2.

## Introduction

COVID-19 is caused by severe acute respiratory syndrome coronavirus 2 (SARS-CoV-2) (Huang et al., 2020). In December 2019, China reported its first case of SARS-CoV-2 in Wuhan, Hubei province (Zhu et al., 2020). The infection was highly contagious, which led to the global spread of the virus in the following months, thus causing an outbreak (Giovanetti et al., 2020; Phan et al., 2020). It was finally declared as a Public Health Emergency of International Concern (PHEIC) by the World Health Organization (WHO) on January 30, 2020, and ultimately named Coronavirus Disease 2019 (COVID-19) on February 12, 2020. COVID-19 is responsible for high mortality worldwide (WHO, 2020a,b). Currently, there are 172, and 61 vaccines are in pre-clinical and clinical development, respectively. While it takes years to develop a new effective drug against a disease, the COVID-19 outbreak has created a global emergency. Hence, drug repositioning and repurposing are given more attention as it is a rapid solution to identify a drug to combat the disease (Li and Clercq, 2020; Morse et al., 2020). Coronaviruses are enveloped viruses about 80–120 nm in diameter with plus-strand (+) RNA as genetic material (Brian and Baric, 2005). Studies have shown that SARS-CoV-2 shares ~80% genetic similarity with SARS-CoV, while RNA-dependent RNA polymerase (RdRp) has a 96% similarity (Xu et al., 2020). RdRp and protease are encoded by the viral RNA and play an essential role in replicating and assembling the virus, and hence preexisting drugs that target these proteins are preferred. Antiviral agents that have been developed to combat coronaviruses are used here as potential drugs for the treatment of COVID-19.

A 20-kb gene encodes the replicase complex. It encompasses several viral genes encoding RdRp, RNA helicase, proteases, and some RNA processing enzymes and cellular proteins that aid in replicating and transcribing the genome of coronavirus occurring in the cytoplasm of the host cell (Koonin and Dolja, 1993; Cheng et al., 2005). RNA synthesis takes place in both a continuous and a discontinuous fashion (Gallagher, 1996). Coronaviruses are plus-strand (+) RNA viruses with a tremendously diverse genome, resulting in a variation in their RNA synthesis machinery (Goldbach, 1987). RdRps exhibit a very high transcription error rate leading to variation in the genome of the virus. During the replication process, RdRps adopts the switching mechanism resulting in the recombination of RNA. This process is also responsible for repairing deleterious mutations in the genome of the viruses, leading to gene rearrangements and new gene acquisition (Strauss and Strauss, 1988; Xu et al., 2003).

Once the virus penetrates the host cell by binding to the angiotensin-converting enzyme 2 (ACE2) receptor, the process of uncoating is initiated that releases the plus-strand (+) RNA into the cytoplasm. The ribosomes present in the host cell's cytoplasm proceed to translate the genomic RNA into a polyprotein. This polyprotein undergoes proteolytic cleavage by the protease enzyme that results in the replicase enzyme production and various viral structural proteins. Approximately 67% of the genomic RNA encodes RdRp that arbitrates the genome's synthesis (Thiel et al., 2001). The plus-strand (+) RNA can only produce viral protein products and not genetic material by replication. Thus, to achieve genome replication, RdRp first transcribes and replicates the plus-strand (+) RNA, which acts as a template to generate the minus-strand (–) RNA, and then serves as a template for the RdRPs to produce several plus-strand (+) RNAs. Some of this plus-strand (+) RNA is again translated into proteins.

**Figure 1:**
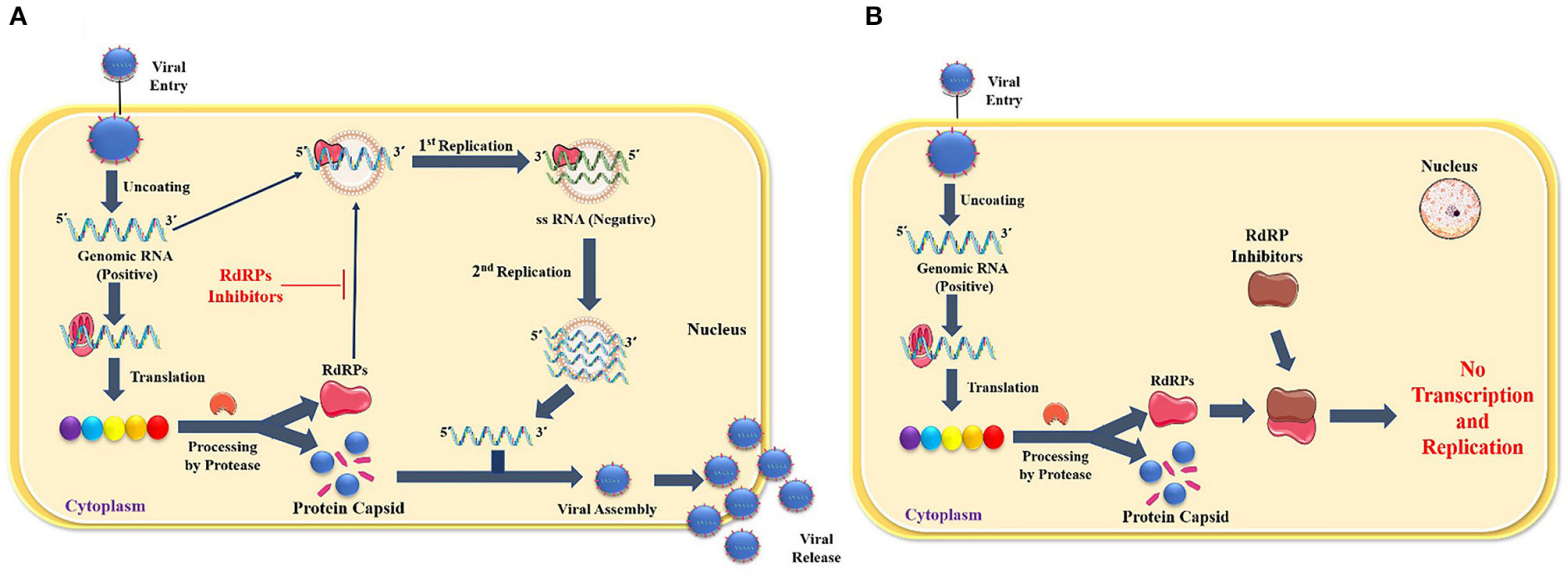
**(A)** Role of RdRp in viral replication, **(B)** Effect of RdRp inhibitors.

In contrast, the others are enclosed into the capsid to regenerate complete virions released from the cell by exocytosis. RdRP is a popular target for a selective antiviral strategy against coronaviruses because RNA synthesis by RdRP does not occur in mammalian cells (Casais et al., 2001). Figure 1 illustrates the role of RdRp in viral replication and effect of RdRp inhibitors (Figure 1).

Current potential treatments against COVID-19 that have shown promising results are Lopinavir, a known protease inhibitor of coronavirus (Yao T.-T. et al., 2020), the guanosine analog, ribavirin that targets RdRp and was designed to combat the Ebola outbreak (Falzarano et al., 2013), and chloroquine, an antimalarial drug, that has shown antiviral effects by blocking viral fusion with the cell due to increased endosomal pH (Vincent et al., 2005). A derivative of chloroquine, hydroxychloroquine, is more potent than chloroquine, as reported by several studies (Yao X. et al., 2020). Several corticosteroids have also been studied for their coronavirus effects (Russell et al., 2020). Convalescent plasma transfusion is also currently being administered and has been shown to reduce mortality rate (Mair-Jenkins et al., 2015). Among the treatments, Remdesivir has shown promising results in *in vitro* and animal studies and is currently in phase III trials (Martinez, 2020).

Remdesivir, also known as GS-5734, is a nucleotide prodrug that is metabolized inside the cell into GS-441524 and an adenosine nucleotide analog, phosphorylated into cell-permeable di-and tri-phosphates (Varga et al., 2016; Warren et al., 2016) Viral RdRps can utilize these adenosine triphosphates during genome replication (Sheahan et al., 2017). Remdesivir causes the nascent RNA transcript to terminate prematurely and incorporates mutations into it (Agostini et al., 2018). Studies have shown that remdesivir causes RNA levels to decrease in a dose-dependent manner, and it is 4.5-fold more potent in cells lacking the ExoN proofreading activity. It has also been observed that GS-5734 is more active (~30 times) than its metabolized form, GS-441524, against the coronaviruses tested (Sheahan et al., 2017). It has been shown that in the presence of GS-441524, there was 5.6-fold resistance in coronavirus mouse hepatitis virus, which was attributed to two mutations, F476L and V553L, in the coding region of nsp12 core polymerase (Agostini et al., 2018). Similar findings were observed in SARS-CoV carrying the mutations F480L and V557L, which are not in the vicinity of the binding site of RdRp; thus, the exact mechanism behind the resistance remains undetermined (Agostini et al., 2018). This raises the concern that resistance may cause the virus to be active and functional, leading to severe disease transmission. Because of its decreased proofreading activity, RdRps have an error rate in the range of 10^**−4**^−10^**−6**^ errors/nucleotide/replication, which is very high compared to that of DNA polymerases (Eckerle et al., 2010). This high error rate is responsible for the rapid rate of virus evolution, contributing to its adaptability to the new surroundings and expediting its ability to jump between species (Pillay and Zambon, 1998). A recent study by Pachetti et al. in 2020 identified the P323L mutation in the RdRp protein of SARS-CoV-2, which may confer resistance to the drug. In this study, we utilized a virtual screening approach to identify the potential repurposed lead compound that might be efficient in treating COVID-19 due to SARS-CoV-2 variants resistant to Remdesivir.

## Materials and Methods

### Remdesivir and COVID19-RdRp Structure Analysis

The 2D and 3D structures of Remdesvir in canonical SMILES and SDF formats were obtained from the PubChem database with CID 121304016. These formats were also converted to PDB using the OpenBabel software (O'Boyle et al., 2011). The 3D structure of the complexes between the non-structural proteins 12, 7, and 8 and Remdesivir was obtained from the Protein Data Bank with PDB ID−7BV2, and the missing amino acids were theoretically built using the Swiss Model Server (Biasini et al., 2014; Wanchao et al., 2020). Further, the P323L mutation reported by Pachetti et al. in 2020 was introduced in the refined protein structure using the SwissPDB Viewer, and energy was minimized using the same method (Guex and Peitsch, 1997; Pachetti et al., 2020).

### Virtual Screening

The inbuilt module of PubChem was used to identify compounds with 90% similarity to the control Remdesivir structure. The identified compounds were screened for absorption, distribution, metabolism, excretion, and toxicity (ADMET) properties using the SwissADME server (Daina et al., 2017). The 90% similar compounds that satisfy the Lipinski drugability properties were further used in an *in silico* antiviral inhibition percentage study using the AntiViral Compound Prediction (AVCpred) server (Qureshi et al., 2017). These filtered compounds were finally subjected to molecular interaction studies against the native and mutant RdRp proteins using the AutoDock standalone package (Morris et al., 2009).

### Molecular Interaction Studies

Molecular interaction studies were initiated to understand the differences between the control remdesivir and the virtually similar compounds with RdRp proteins. Hydrogen and charges were added to the RdRp protein, and the torsions were set to Remdesivir and nearly identical compounds. The grid box was placed around the active site (LYS546, SER683, ARG556, THR688, ASP624, SER760, ASN692, ASP761, and ASP762) retrieved from the published crystal structure (Wanchao et al., 2020). The Lamarckian Genetic Algorithm was used to generate the binding pockets and binding affinity of RdRp for Remdesivir or the virtually similar compounds. One hundred different pockets were developed, and those with the best binding affinity were used in the structural visualization.

### Dynamic Cross-Correlation Matrix (DCCM) and Collective Motion

The docked complexes were further subjected to the web-server DynOmics ENM to identify the residue cross-correlation matrix. The tool developed with the Elastic network models (ENM) that integrate Anisotropic Network Model (ANM) and GaussianNetwork Model (GNM) (Li et al., 2017). Time-correlated data was represented as a matrix between the protein atoms i and j (cij) in DCCM. Within the form of a map, typical fluctuations, and standardized correlations among residues are typically shown and represented with the following equation:

Cij = < ΔRi. ΔRj >
Cij (n) = < ΔRi. ΔRj >/[ < ΔRi. ΔRi > < ΔRj. ΔRj >]1/2

The range of Cij(n) varies in terms (−1, 1) and analyzes information on the cross-correlation between residue movements i and j (Rader et al., 2005). To determine how mutations affect the internal mechanics of protein conformations, the Bio3D module incorporated with the R studio was used to quantify residue-residue dynamic cross-correlation networks. The normal mode, network analysis, and correlation analysis were called with the function “nma(),” “can(),”and “dccm().” The role from these features is usually a cross-correlation matrix of residue-residue. The results were plotted by calling the functions “plot.dccm()” and “pymol.dccm()” (Grant et al., 2006; Scarabelli and Grant, 2013).

## Results

### Virtual Screening

As an initiative of virtual screening, the compounds with 90% structural similarity to the control drug, Remdesivir, were screened using the inbuilt PubChem module. The screening identified 704 compounds possessing 90% similarity with Remdesivir. These 704 compounds were subjected to *in silico* ADME analysis using the SwissADME server (Supplementary Table 1). Based on Lipinski's drugability properties, 32 compounds were further filtered from the pool of 704 compounds. Finally, these 32 compounds were subjected to an antiviral inhibition percentage study calculated using the AVCpred server (Table 1). This analysis identified the compounds with PubChem IDs 137648734, 145074552, 58527341, 44468492, 70649275, 145074438 134502628 that showed higher antiviral inhibition percentages than Remdesivir. The inhibition percentage of Remdesivir was 51.563%. However, the inhibition percentages of compounds, 137648734, 145074552, 58527341, 44468492, 70649275, 145074438, and 134502628 were found to be 62.457, 62.213, 59.052, 57.651, 57.297, 55.189, and 54.648%, respectively. These seven compounds and Remdesivir were subjected to molecular interaction studies against the native and mutant RdRp molecules.

**Table 1:** Antiviral inhibition percentage prediction of the compounds satisfying Lipinski's drugability properties.

**S.No**	**SMILES**	**Ligand**	**PubChem ID**	**General**	**HBV**	**HCV**	**HHV**	**HIV**
1	CCC(CC)COC(=O)C(C)NP(=O)(OCC1C(C(C(O1)(C#N)C2=CC= C3N2N=CN=C3N)O)O)OC4=CC=CC=C4	Remdesvir	121304016	51.563	21.339	54.616	32.307	65.861
2	CCOC(=O)[C@H](C)NP1(=O)OC[C@@H]2[C@@H](O1)[C@@]([C@](O2) (C)C3=CC=C4N3N=CN=C4N)(C)O	44468492	44468492	57.651	21.148	48.128	33.76	62.468
3	C[C@]1([C@@H]([C@H](O[C@@]1(C#N)C2=CC=C3N2N=CN=C3N) COP(=O)(O)OC4=CC=CC=C4)O)F	51041120	51041120	29.001	18.05	45.644	0.293	73.241
4	CCOC(=O)[C@H](C)NP1(=O)OC[C@@H]2[C@@H](O1)[C@@]([C@](O2) (C)C3=CC=C4N3N=CN=C4C)(C)O	58527341	58527341	59.052	20.445	40.745	13.976	61.67
5	CCOC(=O)[C@H](C)NP1(=O)OC[C@@H]2[C@@H](O1)[C@@]3(C[C@]3 (O2)C4=CC=C5N4N=CN=C5N)O	68270743	68270743	43.53	19.756	53.964	40.627	70.965
6	CC(C)C(=O)O[C@@H]1[C@H](O[C@H]([C@]1(C)O)C2=CC=C3N2N =CN=C3N)CO[P+](=O)OC4=CC=CC=C4	70649275	70649275	57.297	20.377	33.6	23.985	61.671
7	C[C@]1([C@@H]([C@H](O[C@@]1(C#N)C2=CC=C3N2N=CN=C3N) CO[P+](=O)OC4=CC=CC=C4)O)O	90048786	90048786	39.527	17.712	55.339	17.268	61.24
8	C[C@@]1([C@@H]([C@@H]([C@H](O1)COP(=O)(N)OC2=CC=CC= C2)O)O)C3=CC=C4N3N=CN=C4N	117913880	117913880	49.781	18.567	24.086	51.373	63.478
9	C[C@@]1([C@@H]([C@@H]([C@H](O1)COP(=O)(N)OC2=CC=CC= C2)O)O)C3=CC=C4N3N=CN=C4N=NN	117913912	117913912	51.369	17.632	55.849	32.075	74.323
10	C[C@@H](C(=O)OC1CCC1)NP(=O)(C)OC[C@@H]2[C@H]([C@H]([C@] (O2)(C#N)C3=CC=C4N3N=CN=C4N)O)O	121310126	121310126	43.53	19.756	53.964	40.627	70.965
11	C1=CC=C(C=C1)OP(=O)(O)OC[C@@H]2[C@H]([C@H]([C@] (O2)(C#N)C3=CC=C4N3N=CN=C4N)O)O	121313145	121313145	43.449	20.677	51.671	41.029	73.631
12	CCC(CC)COC(=O)[C@H](C)N[P+](=O)OC[C@@H]1CC[C@](O1) (C#N)C2=CC=C3N2N=CN=C3N	126693021	126693021	30.141	11.579	53.901	30.889	66.354
13	C[C@@]1([C@@H]([C@@H]([C@H](O1)CO[P+](=O)OC2=CC= CC=C2)O)O)C3=CC=C4N3N=CN=C4N	126719084	126719084	45.799	21.359	48.486	20.775	66.338
14	C[C@@H](C(=O)OCC(C)(C)C)N[P+](=O)OC[C@@H]1[C@H]([C@H] ([C@](O1)(C#N)C2=CC=C3N2N=CN=C3N)O)O	126719091	126719091	29.001	18.05	45.644	0.293	73.241
15	C[C@@H](C(=O)OC)N[P+](=O)OC[C@@H]1[C@H]([C@H]([C@] (O1)(C#N)C2=CC=C3N2N=CN=C3N)O)O	126719092	126719092	35.15	19.336	67.523	42.404	62.287
16	C[C@@]1([C@@H]([C@@H]([C@H](O1)COP(=O)(O)OC2=CC= CC=C2)O)O)C3=CC=C4N3N=CN=C4N	130312728	130312728	23.45	23.058	21.02	29.107	63.229
17	CCC(CC)COC(=O)CN[P+](=O)OC[C@@H]1[C@H]([C@H] ([C@](O1)(C#N)C2=CC=C3N2N=CN=C3N)O)O	130312749	130312749	37.635	20.432	52.161	59.727	60.1
18	CCC(CC)COC(=O)C(C)NP(OCC1CCC(O1)C2=CC=C3N2N=CN= C3N)OC4=CC=CC=C4	132046034	132046034	45.799	21.359	48.486	20.775	66.338
19	CCC(CC)COC(=O)[C@H](C)N[P+](=O)OC[C@@H]1C[C@H] ([C@](O1)(C#N)C2=CC=C3N2N=CN=C3N)O	134443511	134443511	30.21	13.353	53.898	30.99	66.363
20	C[C@@]1([C@@H]([C@@H](C(O1)CO[P+](=O)OC2=CC=CC =C2)O)O)C3=CC=C4N3N=CN=C4N	134502618	134502618	26.991	20.168	68.022	44.334	72.874
21	C1=CC=C(C=C1)O[P+](=O)OC[C@@H]2[C@H]([C@H] ([C@](O2)(C#N)C3=CC=C4N3N=CN=C4N)O)O	134502627	134502627	30.818	20.362	67.286	38.195	65.452
22	CC(C(=O)O)NP(=O)(O)OC[C@@H]1C[C@H]([C@](O1)(C#N) C2=CC=C3N2N=CN=C3N)O	134502628	134502628	54.648	17.777	54.79	40.625	62.179
23	CCOC(=O)[C@H](C)NP(=O)(O)OC[C@@H]1[C@H]([C@@]([C@] (O1)(C#N)C2=CC=C3N2N=CN=C3N)(C)O)O	137648734	137648734	62.457	20.55	48.165	33.256	63.195
24	C[C@@H](C(=O)OC(C)C)NP(=O)(O)OC[C@@H]1[C@H]([C@@] ([C@](O1)(C#N)C2=CC=C3N2N=CN=C3N)(C)O)O	137660077	137660077	40.854	20.469	13.066	18.678	67.667
25	CCC(CC)COC(=O)[C@H](C)N[P+](=O)OC[C@@H]1[C@H] (C[C@](O1)(C#N)C2=CC=C3N2N=CN=C3N)O	138525664	138525664	18.701	20.275	56.884	28.924	73.168
26	C1=CC=C(C=C1)OP(=O)(O)OCC2[C@H]([C@H]([C@](O2) (C#N)C3=CC=C4N3N=CN=C4N)O)O	138525701	138525701	26.651	20.42	26.507	38.09	68.021
27	CC(C)OC(=O)CNP(OC[C@@H]1C[C@@H]([C@@H](O1)C2=CC= C3N2N=CN=C3N)C#N)OC4=CC=CC=C4	138529102	138529102	39.45	19.357	62.986	54.581	79.639
28	CCOC(=O)CNP(OC[C@@H]1C[C@@H]([C@@H](O1)C2=CC= C3N2N=CN=C3N)C#N)OC4=CC=CC=C4	138529141	138529141	35.048	20.109	62.385	38.05	69.065
29	C[C@]1([C@H]([C@H]([C@@H](O1)C2=CC=C3N2N=CN= C3N)O)O)CO[P+](=O)OC4=CC=CC=C4	138598986	138598986	47.712	20.613	58.371	33.193	64.964
30	CN=C[C@@]1([C@@H]([C@@H](C(O1)COP(=O)(O)OC2=CC= CC=C2)O)O)C3=CC=C4N3N=CN=C4N	139476292	139476292	22.199	23.047	21.248	13.219	61.262
31	CCC(CC)COC(=O)CN[P@](OCC1CCC(O1)C2=CC=C3N2N=CN =C3N)OC4=CC=CC=C4	145074438	145074438	55.189	20.709	49.992	33.319	63.255
32	CCC(CC)COC(=O)C(C)N[P@](OCC1CCC(O1)C2=CC=C3N2N= CN=C3N)OC4=CC=CC=C4	145074498	145074498	39.424	19.374	62.987	54.504	79.64
33	CC(C)OC(=O)CNP(OCC1CCC(O1)(C#N)C2=CC=C3N2N=CN =C3N)OC4=CC=CC=C4	145074552	145074552	62.213	21.225	48.134	33.256	66.604

### Molecular Interaction Studies

**Figure 2:**
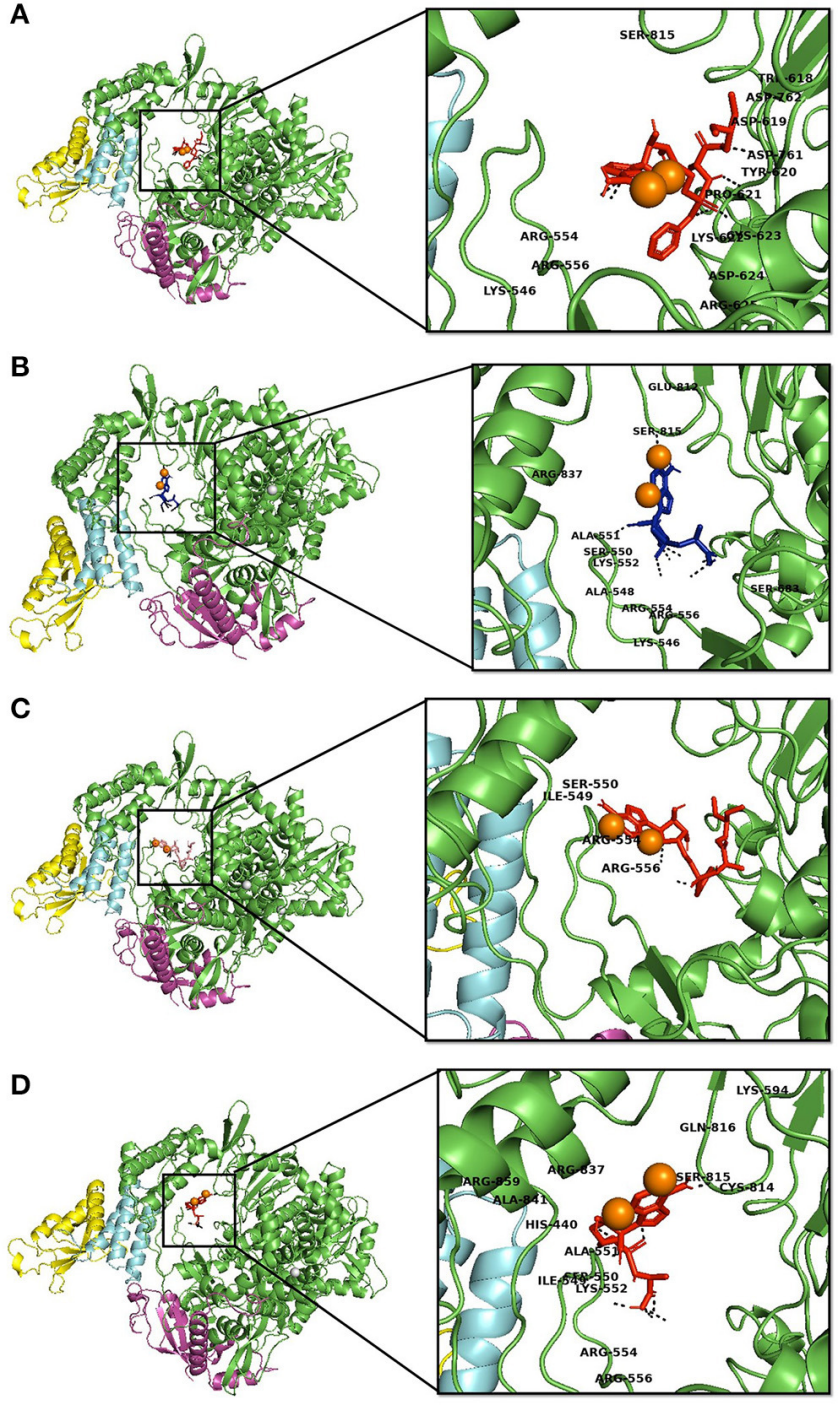
Interaction of Remdesvir and the novel lead compound with the native RdRp and RdRp with the mutation. **(A)** Native RdRp with Remdesvir, **(B)** Native RdRp with novel lead compound (SCHEMBL20144212), **(C)** Mutant RdRp with Remdesvir, **(D)** Mutant RdRp with novel lead compound (SCHEMBL20144212).

Molecular interaction studies were performed using AutoDock to understand the interaction pattern of Remdesivir and its similar compounds with the native and mutant RdRp proteins. It was observed that all seven compounds had a higher binding affinity than Remdesivir with native RdRp. For RdRp protein carrying the P323L mutation, we observed that the compounds 134502628, 70649275, 137648734, and 145074552 possessed higher binding affinity than Remdesivir. The binding affinity of Remdesivir for native and mutant RdRp was −4.5 and −4.2 kcal/mol, respectively. However, the compound with PubChem ID 134502628 showed the highest binding affinity of −7.5 and −7.4 kcal/mol for the native and mutant RdRp proteins, respectively (Tables 2A,B). The detailed interaction comparison between Remdesivir and top-ranked compound, 134502628 (SCHEMBL20144212), against the native and mutant RdRp is shown in Table 3, Figures 2A–D.

**Table 2:** The binding affinity of the ligands against native and mutant SARS-CoV-2 RdRp structures.

**S.No**	**Protein**	**Ligands**	**Binding Affinity** ** (kcal/mol)**
**(A)**			
1	Native RdRp	134502628	−7.5
2	Native RdRp	58527341	−6.3
3	Native RdRp	145074552	−6
4	Native RdRp	145074438	−6
5	Native RdRp	137648734	−5.5
6	Native RdRp	70649275	−5.3
7	Native RdRp	44468492	−5.1
8	Native RdRp	Remdesvir	−4.5
**(B)**			
1	RdRp with P323L	134502628	−7.4
2	RdRp with P323L	70649275	−6.6
3	RdRp with P323L	137648734	−6.3
4	RdRp with P323L	145074552	−6.1
5	RdRp with P323L	Remdesvir	−5.6
6	RdRp with P323L	145074438	−5.5
7	RdRp with P323L	44468492	−5.3
8	RdRp with P323L	58527341	−4.2

***(A)** Against native RdRp, **(B)** against mutant RdRp*.

**Table 3:** Differences in the binding affinity (kcal/mol), number of hydrogen bonds, number of interacting amino acids, and Interacting amino acids between native and mutant RdRp against Remdesivir and the novel lead compound.

**S.No**	**Protein**	**Ligands**	**Binding affinity** ** (kcal/mol)**	**Number of hydrogen bonds**	**Number of interacting amino acids**	**Interacting amino acids**
1	Native RdRp	Remdesivir	−4.5	6	14	SER815, TRP618, ASP762, ASP619, ASP761, TYR620, PRO621, LYS622, LYS623, ASP624, ARG625, ARG554, ARG556, LYS546
2	Native RdRp	SCHEMBL20144212	−7.5	7	11	GLU812, SER815, ARG837, ALA551, SER550, LYS552, ALA548, ARG554, ARG556, LYS546, SER683
3	RdRp with P323L mutation	Remdesivir	−4.2	2	4	SER550, ILE549, ARG554, ARG556
4	RdRp with P323L mutation	SCHEMBL20144212	−7.4	6	14	LYS594, GLN816, SER815, CYS814, ARG837, ARG859, ALA841, HIS440, ALA551, SER550, ILE549, LYS552, ARG554, ARG556

### DCCM and Collective Motion

**Figure 3:**
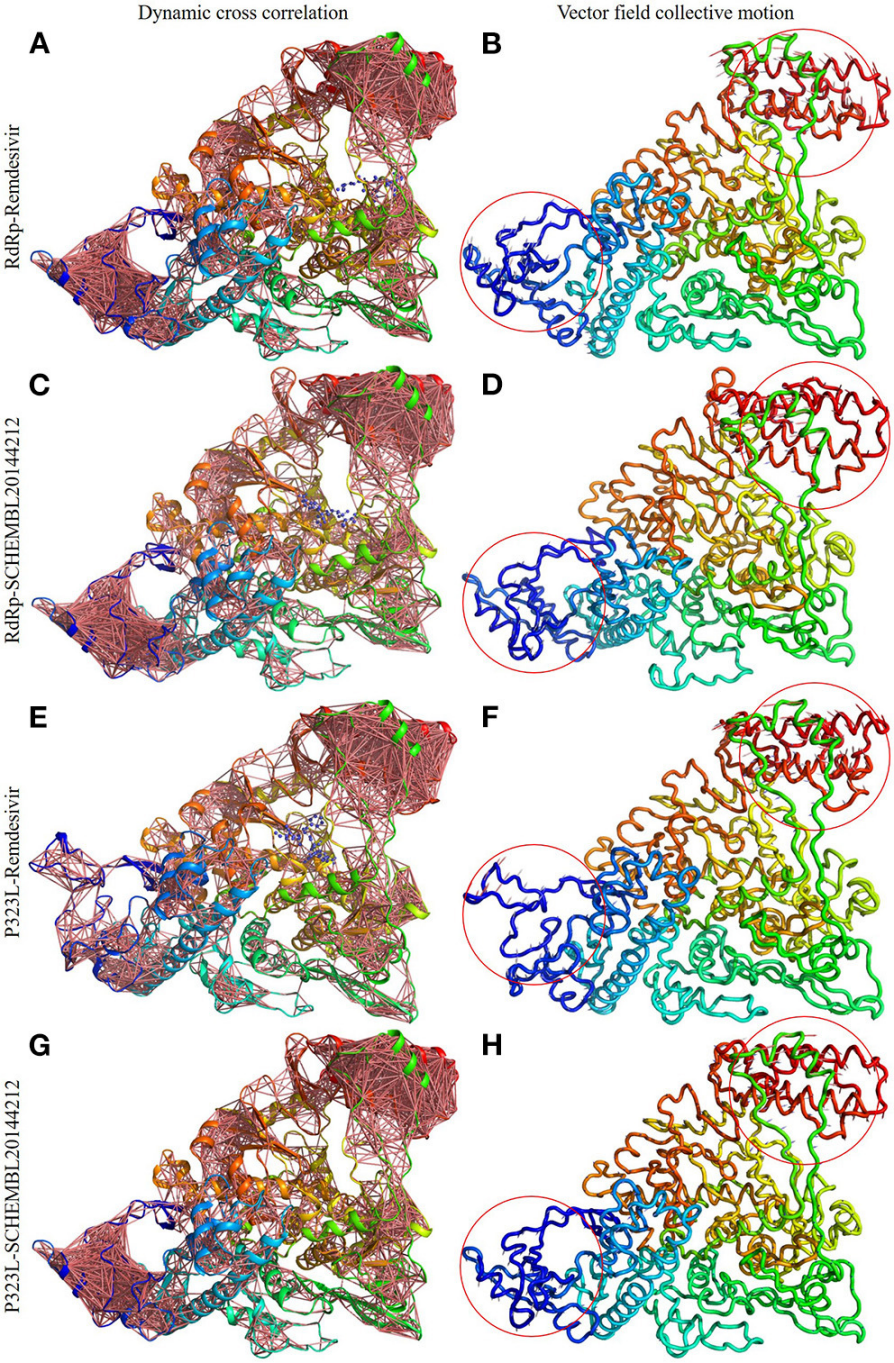
Dynamic cross-correlation networks and vector field collective motions of docked complexes. Depiction of cross-correlation networks: **(A)** RdRp-Remdesivir; **(C)** RdRp-SCHEMBL20144212; **(E)** P323L-Remdesivir; **(G)** P323L-SCHEMBL20144212. Depiction of vector field collective motions: **(B)** RdRp-Remdesivir; **(D)** RdRp-SCHEMBL20144212; **(F)** P323L-Remdesivir; **(H)** P323L-SCHEMBL20144212. Red circles indicate differences in the direction of collective motions by arrows.

**Figure 4:**
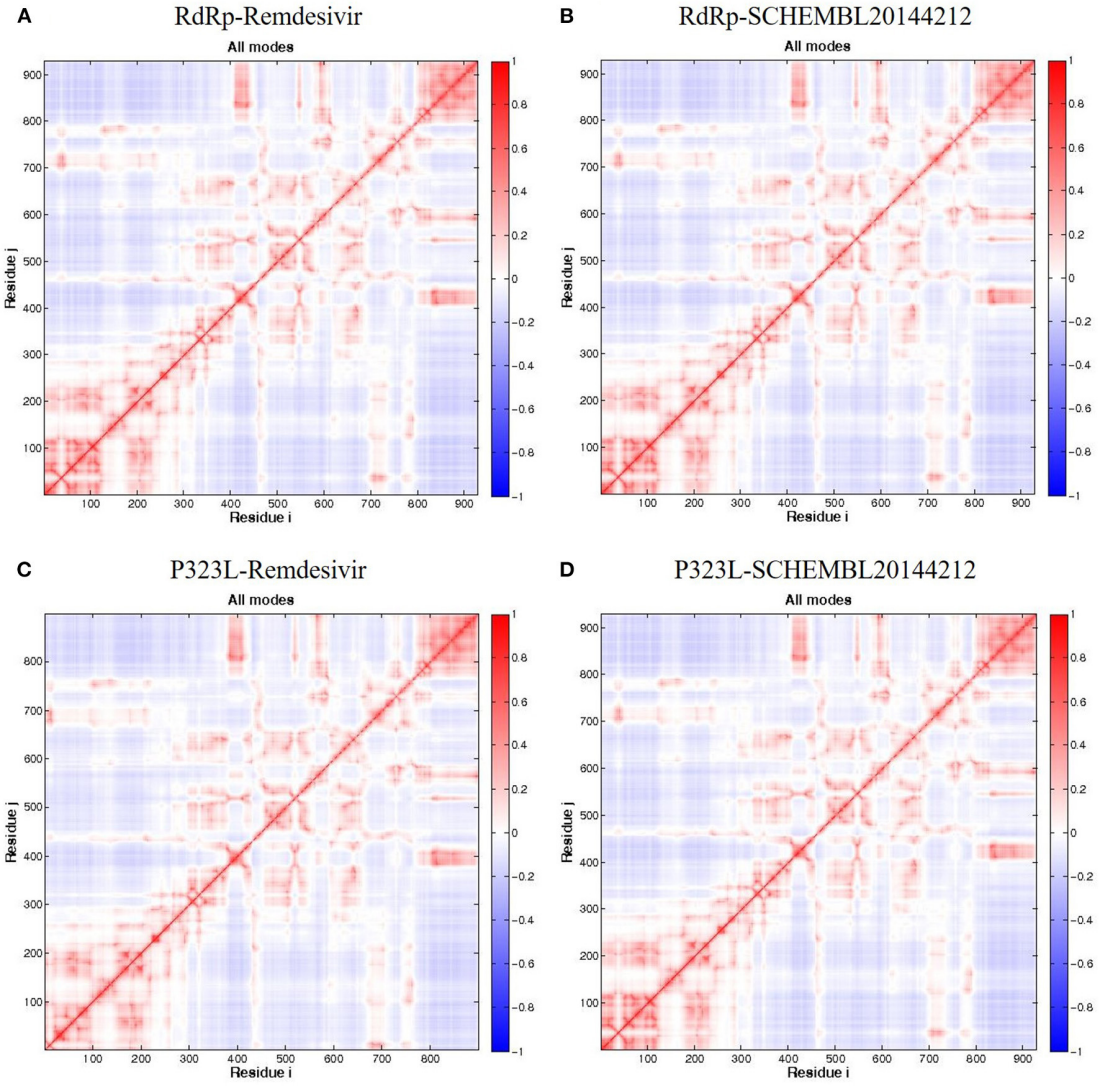
DCCM of the residues around their mean position for all docked RdRp complexes. **(A)** RdRp-Remdesivir; **(B)** RdRp-SCHEMBL20144212; **(C)** P323L-Remdesivir; **(D)** P323L-SCHEMBL20144212. Map size = 100, min i = 5, and min j = 5. The color ranges from −1 (blue) to +1 (red), whereas 0 remains in white color.

Firstly, we investigated the residue cross-correlation networks and vector field collective motions with the help of the Bio3D module that was integrated within R studio. All four complexes were subjected to the Bio3D module to retrieve the residue cross-correlation networks and collective motions. As a result, the residues from 5 to 120 (α1, α2, α3, β1, β2, and β-hairpin) and 830–933 (α40–45, β22, and β23) were obtained with large cross-correlation networks from RdRp-Remdesivir and SCHEMBL20144212 complexes (Figures 3A,C), whereas the P323L-Remdesivir obtained less cross-correlation networks especially at the region from 5 to 120 residues (Figure 3E). The residues from 830 to 933 exhibited similar networks for all the docked complexes. However, the differences can be seen with the P323L-Remdesivir and P323L-SCHEMBL20144212 complexes, especially at the region from 5 to 120 residues. The P323L-Remdesivir complex exhibited the least residue cross-correlation; the possible reason for this could be mutation at 323rd position in RdRp. Our results exhibited similar network cross-correlation for P323L-SCHEMBL20144212 (Figure 3G) when compared to the RdRp-SCHEMBL20144212 complex. The atomic movements of these docked complexes were investigated by representing the vector field collective motions. The collective motions were largely observed in the 5–120 and 830–933 regions, as seen in Figures 3B,D,F,H. Here, we observed the differences in the residues from 5 to 120 from the P323L-Remdesivir complex (Figure 3F) compared to the other three docked complexes. The arrows on the docked complex of proteins determine the magnitude and direction of the collective motion. Furthermore, we investigated the DCCM of RdRp (with or without mutation) with remdesivir and SCHEMBL20144212 complexes by utilizing the DynOmics tool. DCCM explores the precise movements of residues of proteins, demonstrates strongly correlated (in red) atomic movements between residues, and highlights atomic movements that are strongly anticorrelated (in blue) (Figure 4). The RdRp-SCHEMBL20144212 and P323L-SCHEMBL20144212 complexes (Figures 4B,C) illustrate the strongly correlated motions and less anticorrelation as compared to that of RdRp-Remdesivir and P323L-Remdesivir complexes (Figures 4A,C).

## Discussion

COVID-19, caused by SARS-CoV-2, has become a global pandemic and is responsible for about 2,71,954 deaths worldwide, with more than 3.95 million positive cases. COVID-19 has been transmitted to almost 120 countries, and discovering drugs to fight this disease is a great challenge (Worldometer, 2021). The genome of the SARS-CoV-2 virus was immediately sequenced to improve our understanding of the virus, formulate appropriate diagnostic and preventive techniques, and develop therapeutic strategies (Wang et al., 2020). The high transmission rate of the virus is responsible for the difficulty of implementing efficient preventive measures. Hence, there is an immediate requirement for a drug to inhibit the virus and eliminate the disease (Li et al., 2020). One of the emerging drugs with promising results is Remdesivir. It is a nucleotide analog, initially developed for the Ebola virus, that targets the virus's RdRp (Gordon et al., 2020). The virus's high mutation rate may introduce changes to RdRp, rendering the drug ineffective (Eckerle et al., 2010). A recent study by Pachetti et al. in 2020 identified the P323L mutation in the RdRp of SARS-CoV-2 (Pachetti et al., 2020). The possible increase in mutations highlights the need to develop a series of drugs whose efficiency is not affected by these mutations.

Thus, there is an urgent need to develop novel drugs. The conventional method for drug discovery is expensive and time-consuming, and therefore the *in silico* approach is a means to move forward. Our study formulated a computational pipeline to identify a series of compounds similar to Remdesivir, which could be used as an alternative if SARS-CoV-2 develops resistance to Remdesivir due to mutations. Out of 704 compounds, which were 90% similar to Remdesivir, 32 compounds were found to satisfy the rule of 5 (Supplementary Table 1). To assess drugability, these 32 compounds were subjected to *in silico* antiviral inhibition percentage analysis. We observed that the seven virtually similar compounds possessed a higher antiviral inhibition percentage when compared to Remdesivir. Finally, molecular interaction studies against the native and mutant RdRp were carried out with docking analysis. The interactions revealed that all the identified seven compounds could interact with higher affinity with the native protein than Remdesivir.

However, upon introducing the P323L mutation, only four compounds were found to bind with a higher binding affinity than Remdesivir. The binding efficacy of Remdesivir was also found to decrease from −4.5 kcal/mol in the native to −4.2 kcal/mol in the mutant protein. Interestingly, the compound, SCHEMBL20144212, was topmost in the binding affinity for native and mutant proteins (Table 2). Remdesivir was found to interact with the native RdRp through 14 amino acids and 6 hydrogen bonds. However, the identified novel lead compound, SCHEMBL20144212, was found to interact with native RdRp through 11 amino acids and 7 hydrogen bonds.

Similarly, in interaction with the mutant protein, Remdesivir was found to interact through four amino acids and two hydrogen bonds. However, the novel lead compound, SCHEMBL20144212, was found to interact through 14 amino acids and 6 hydrogen bonds (Table 3, Figures 2A–D). The role of hydrogen bonds and interacting amino acids is crucial for a drug to exhibit its efficacy. We observed higher binding affinity, hydrogen bond interactions, and amino acid interactions with the identified novel lead compound compared to Remdesivir. We have also depicted the detailed pharmacokinetic properties obtained from the pkCSM server in Table 4. These results suggest that SCHEMBL20144212 is more effective against the original or the mutated virus compared with Remdesivir.

**Table 4:** ADMET properties of the novel lead compound obtained from the pkCSM server.

**Property**	**Model name**	**Predicted value**	**Unit**
Absorption	Water solubility	−2.347	Numeric (log mol/L)
Absorption	Caco2 permeability	−0.496	Numeric (log Papp in 10–6 cm/s)
Absorption	Intestinal absorption (human)	28.391	Numeric (% Absorbed)
Absorption	Skin Permeability	−2.735	Numeric (log Kp)
Absorption	P–glycoprotein substrate	No	Categorical (Yes/No)
Absorption	P-glycoprotein I inhibitor	No	Categorical (Yes/No)
Absorption	P-glycoprotein II inhibitor	No	Categorical (Yes/No)
Distribution	VDss (human)	−0.036	Numeric (log L/kg)
Distribution	Fraction unbound (human)	0.546	Numeric (Fu)
Distribution	BBB permeability	−1.906	Numeric (log BB)
Distribution	CNS permeability	−4.432	Numeric (log PS)
Metabolism	CYP2D6 substrate	No	Categorical (Yes/No)
Metabolism	CYP3A4 substrate	No	Categorical (Yes/No)
Metabolism	CYP1A2 inhibitor	No	Categorical (Yes/No)
Metabolism	CYP2C19 inhibitor	No	Categorical (Yes/No)
Metabolism	CYP2C9 inhibitor	No	Categorical (Yes/No)
Metabolism	CYP2D6 inhibitor	No	Categorical (Yes/No)
Metabolism	CYP3A4 inhibitor	No	Categorical (Yes/No)
Excretion	Total Clearance	0.179	Numeric (log ml/min/kg)
Excretion	Renal OCT2 substrate	No	Categorical (Yes/No)
Toxicity	AMES toxicity	No	Categorical (Yes/No)
Toxicity	Max. tolerated dose (human)	0.643	Numeric (log mg/kg/day)
Toxicity	hERG I inhibitor	No	Categorical (Yes/No)
Toxicity	hERG II inhibitor	No	Categorical (Yes/No)
Toxicity	Oral Rat Acute Toxicity (LD50)	2.376	Numeric (mol/kg)
Toxicity	Oral Rat Chronic Toxicity (LOAEL)	2.307	Numeric (log mg/kg_bw/day)
Toxicity	Hepatotoxicity	Yes	Categorical (Yes/No)
Toxicity	Skin Sensitisation	No	Categorical (Yes/No)
Toxicity	T.Pyriformis toxicity	0.285	Numeric (log ug/L)
Toxicity	Minnow toxicity	3.311	Numeric (log mM)

It is vital to characterize the protein folding via identifying conformational variances, change in conformational motions owing to mutations, mechanism of ion channel's opening/closing, and protein-ligand binding, as these factors directly related to the protein's stability and function (Grottesi et al., 2005; Yang et al., 2008; Kim et al., 2010; S et al., 2020; Udhaya Kumar et al., 2020). The residue cross-correlation networks were obtained for all the docked complexes, and among them, the P323L-Remdesivir complex obtained less correlative network at the region from 5 to 120 residues (Figure 3E). A possible reason for this could be a mutation that occurred at position 323; thus, complex resulted in fewer correlative networks. Also, in comparison to our identified lead compound (SCHEMBL20144212) with mutant RdRp (P323L) exhibited large correlative networks and could act as a sturdy inhibitor against mutant RdRp (Figure 3G). The vector field collective motions were identified for all the docked complexes to explore each complex's dynamic motion and magnitude (Figures 3B,D,F,H). The RdRp and mutant RdRp (P323L) with SCHEMBL20144212 lead compound exhibited a rigid complex as seen in Figures 3D,H. This clearly states that the SCHEMBL20144212 lead compound inhibits the RdRp from binding to other molecules and for the catalysis process, whereas the remdesivir complexes exhibited more flexible regions (Figures 3B,F) (Huber, 1987; Karshikoff et al., 2015). Furthermore, our results from DCCM showed the RdRp-SCHEMBL20144212 and P323L-SCHEMBL20144212 complexes exhibited strongly correlated motions (Figures 4B,D). From the above observations, we strongly believe that the molecular docking of SCHEMBL20144212 lead compound reduces the effect of P323L mutation and could act as an effective inhibitor against SARS-nCoV-2's RdRp.

## Conclusion

This study was initiated to identify the potential repurposed lead compound to treat COVID-19 in case of possible SARS-CoV-2-virus resistance to Remdesivir due to mutations. The drug inhibits the activity of RdRp protein to a small extent. Identifying the mutation in RdRp of SARS-CoV-2 highlights the possible appearance of mutated viruses that might be resistant to current and future treatments. Therefore, it is necessary to establish a platform to identify compounds similar to but more useful than Remdesivir. Our study identified a total of 704 compounds from the PubChem database, which are 90% similar to Remdesivir. Of these 704 compounds, 32 compounds were found to possess druggability properties, out of these, seven compounds were found to possess higher antiviral inhibition percentages when compared to Remdesivir. Out of these seven compounds analyzed for molecular interaction. The SCHEMBL20144212 compound was found to possess the highest interaction affinity for both the native and mutant RdRp protein. From the DCCM and vector field collective motion analysis, we demonstrated that our lead compound's molecular docking (SCHEMBL20144212) tolerates the effect of the P323L mutation and could act as an effective inhibitor against SARS-nCoV-2's RdRp. Thus, this compound should be further validated through *in vitro* experiments as an alternative to Remdesivir to bypass the resistant effect of the P323L mutation. Our study offers a platform for drug repurposing during the time of viral pandemics.

## Data Availability Statement

The original contributions presented in the study are included in the article/Supplementary Material, further inquiries can be directed to the corresponding author/s.

## Author Contributions

TK, UK, HZ, and GD contributed to designing the study and data acquisition, analysis, and interpretation. TK, NS, and UK involved in the acquisition, analysis, and interpreting of the results. TK, UK, and GD contributed to data interpretation, conducted, and drafting the manuscript. GD and HZ supervised the entire study and were involved in study design, acquiring, analyzing, understanding the data, and drafting the manuscript. The manuscript was reviewed and approved by all the authors.

## Conflict of Interest

The authors declare that the research was conducted in the absence of any commercial or financial relationships that could be construed as a potential conflict of interest.

## Supplementary Material

Supplementary Table 1 The list of 90% similar compounds to the Remdesivir obtained from the PubChem database.
